# Impact of Taxanes, Endocrine Therapy, and Deleterious Germline *BRCA* Mutations on Anti-müllerian Hormone Levels in Early Breast Cancer Patients Treated With Anthracycline- and Cyclophosphamide-Based Chemotherapy

**DOI:** 10.3389/fonc.2019.00575

**Published:** 2019-07-12

**Authors:** Matteo Lambertini, Nathalie Olympios, Justine Lequesne, Céline Calbrix, Maxime Fontanilles, Agnès Loeb, Marianne Leheurteur, Isabelle Demeestere, Frédéric Di Fiore, Anne Perdrix, Florian Clatot

**Affiliations:** ^1^Department of Medical Oncology, U.O.C. Clinica di Oncologia Medica, IRCCS Ospedale Policlinico San Martino, Genova, Italy; ^2^Department of Internal Medicine and Medical Specialties, School of Medicine, University of Genova, Genova, Italy; ^3^Department of Medical Oncology, Centre Henri Becquerel, Rouen, France; ^4^Department of Clinical Research and Biostatistics, Centre Henri Becquerel, Rouen, France; ^5^Department of Bio-Pathology, Centre Henri Becquerel, Rouen, France; ^6^IRON Group, UNIROUEN, Inserm U1245, Normandy Centre for Genomic and Personalized Medicine, Rouen University Hospital, Normandie University, Rouen, France; ^7^Department of Medical Information, Henri Becquerel Centre, Rouen, France; ^8^Research Laboratory on Human Reproduction, Fertility Clinic, CUB-Hôpital Erasme and Université Libre de Bruxelles, Brussels, Belgium

**Keywords:** breast cancer, AMH, taxane, endocrine therapy, *BRCA* mutations

## Abstract

**Background:** Limited evidence exists on the impact of adding a taxane, using endocrine therapy and carrying a deleterious germline *BRCA* mutation on ovarian reserve measured by anti-müllerian hormone (AMH) levels of young breast cancer patients receiving (neo)adjuvant cyclophosphamide- and anthracycline-based chemotherapy.

**Methods:** This is a biomarker analysis including young (≤ 40 years) early breast cancer patients with known germline *BRCA* mutational status and available prospectively collected frozen plasma samples before and after chemotherapy. Chemotherapy consisted of either six cycles of FEC (5 fluorouracil 500 mg/m^2^, epirubicin 100 mg/m^2^, cyclophosphamide 500 mg/m^2^) or three cycles of FEC followed by three cycles of docetaxel (D, 100 mg/m^2^). Endocrine therapy consisted of tamoxifen (±GnRH agonists). AMH levels at baseline, 1 and 3 years after diagnosis were compared according to type of chemotherapy (FEC only vs. FEC-D), use of endocrine therapy (yes vs. no) and deleterious germline *BRCA* mutations (mutated vs. negative).

**Results:** Out of 148 included patients, 127 (86%) received D following FEC chemotherapy, 90 (61%) underwent endocrine therapy, and 35 (24%) had deleterious germline *BRCA* mutations. In the whole cohort, AMH levels drastically dropped 1 year after diagnosis (*p* < 0.0001) with a slight but significant recovery at 3 years (*p* < 0.0001). One year after diagnosis, patients treated with FEC only had higher median AMH levels than those who received FEC-D (0.22 vs. 0.04 μg/L, *p* = 0.0006); no difference was observed at 3 years (0.06 and 0.18 μg/L, *p* = 0.47). Patients under endocrine therapy had significantly higher AMH levels than those who did not receive this treatment 1 year after diagnosis (0.12 vs. 0.02 μg/L; *p* = 0.008), with no difference at 3 years (0.11 and 0.20 μg/L, *p* = 0.22). AMH levels were similar between *BRCA*-mutated and *BRCA*-negative patients at baseline (1.94 vs. 1.66 μg/L, *p* = 0.53), 1 year (0.09 vs. 0.06 μg/L, *p* = 0.39) and 3 years (0.25 vs. 0.16 μg/L; *p* = 0.43) after diagnosis.

**Conclusions:** In breast cancer patients receiving FEC chemotherapy, adding D appeared to negatively impact on their ovarian reserve in the short-term; no further detrimental effect was observed for endocrine therapy use and presence of a deleterious germline *BRCA* mutation.

## Introduction

As a consequence of the higher incidence of aggressive tumor subtypes and the negative prognostic value of young age at diagnosis (1, 2), a significant proportion of young women with early breast cancer are candidates to receive adjuvant or neoadjuvant chemotherapy. In young patients, a major potential drawback associated with the use of systemic cytotoxic therapy is represented by the risk of causing gonadal damage with subsequent premature ovarian insufficiency (POI) and infertility (3). Considering the substantial quality of life implications associated with the development of these side effects, appropriate oncofertility counseling is now considered mandatory with all cancer patients diagnosed during their reproductive years (4–6).

Age and use of cyclophosphamide-based chemotherapy are the two major known determinants influencing the risk of gonadal damage following the use of anticancer treatments in young women with breast cancer (3). On the contrary, the impact of other treatment- or patient-related factors remains controversial. Specifically, while the effect of cyclophosphamide- and anthracycline-based chemotherapy regimens is well-established, it remains unclear if the addition of a taxane can further increase the risk of gonadal damage (7). Similarly, in young patients with hormone receptor-positive disease, the gonadotoxic impact of using endocrine therapy following chemotherapy remains debated (7, 8). Finally, recent evidence suggests that carrying a deleterious germline *BRCA* mutation may have a negative impact on women ovarian reserve raising the important concern of a potential increased gonadotoxicity with the use of anticancer treatments in this patient population (9, 10).

Informing young women with newly diagnosed breast cancer about the actual gonadal damage associated with the use of the proposed anticancer treatments is even more complex considering that the majority of the studies that addressed this issue relied only on the presence or absence of menstrual function after the end of chemotherapy (3). However, this cannot be considered a surrogate to determine treatment-induced gonadotoxicity (11). Being the most accurate indicator of remaining ovarian reserve, anti-müllerian hormone (AMH) is considered a promising biomarker of treatment-induced gonadotoxicity (12). Nevertheless, to date, there is limited evidence on the actual gonadal damage associated with the use of the proposed anticancer treatments through AMH assessment in young breast cancer patients (12). This is crucial information to be acquired to better inform these patients about the adverse events associated with the proposed therapies as well as on the need of pursuing fertility preservation strategies before treatment initiation. To acquire more insights on this unmet medical issue, we conducted the present study aiming to evaluate the impact of adding a taxane, using endocrine therapy as well as carrying a deleterious germline *BRCA* mutation on the ovarian reserve measured by AMH levels at baseline and up to 3 years after diagnosis in young early breast cancer patients treated with (neo)adjuvant anthracycline- and cyclophosphamide-based chemotherapy.

## Materials and Methods

### Patients and Treatments

This is a biomarker analysis conducted within a cohort of consecutive patients with early breast cancer diagnosed at ≤ 40 years that underwent (neo)adjuvant chemotherapy between January 2008 and December 2016 at the Henri Becquerel Cancer Center (Rouen, France). Patients with known germline *BRCA* mutational status and prospectively collected and available frozen plasma samples before and after chemotherapy were eligible for inclusion in the present analysis.

Chemotherapy consisted of either six cycles of FEC (five fluorouracil 500 mg/m^2^, epirubicin 100 mg/m^2^, cyclophosphamide 500 mg/m^2^) or three cycles of FEC followed by three cycles of docetaxel (D, 100 mg/m^2^). Adjuvant endocrine therapy consisted of tamoxifen exclusively or associated with gonadotropin-releasing hormone (GnRH) agonists. None of the patients received GnRH agonists during chemotherapy for ovarian function and/or fertility preservation.

All patients signed a consent form allowing the conservation and study of their biological samples. The present study was approved by the Institutional Scientific and Ethics Committees of Henri Becquerel Centre (registering order N°1807B).

### AMH Measurements

As per routine practice at our center, plasma samples are prospectively collected at fixed timepoints during follow-up in all breast cancer patients and are stored in our plasma bank at −20°C.

For the purpose of the present analysis, plasma samples of eligible patients were used to assess AMH levels at baseline (i.e., before starting chemotherapy), ~1 year and over 3 years after diagnosis.

AMH measurements were centrally performed at Henri Becquerel Cancer Center using fully automated ultra-sensitive Elecsys AMH assay on the Cobas e601 instrument (Roche Diagnostics). The detection and quantification limits were 0.01 and 0.03 μg/L, respectively, with an intra-assay imprecision coefficient of variation equal to 1.2% at 1.19 μg/L and 0.9% at 5.89 μg/L.

### Study Objectives

The main objective of this study was to assess the impact of adding a taxane (D), using endocrine therapy and carrying a deleterious germline *BRCA* mutation on the ovarian reserve measured by AMH levels of young breast cancer patients after (neo)adjuvant cyclophosphamide- and anthracycline-based chemotherapy (FEC). AMH levels at baseline, 1 and 3 years after diagnosis were compared according to type of chemotherapy (FEC only vs. FEC-D), use of endocrine therapy (yes vs. no) and deleterious germline *BRCA* mutations (mutated vs. negative).

### Statistical Analysis

Baseline characteristics of patients were compared according to type of chemotherapy or endocrine treatment administered and *BRCA* mutational status. Quantitative variables were reported as median with interquartile range (IQR, Q1–Q3). Differences were tested using χ^2^, Fisher's exact test or two-sample *t*-tests as appropriate.

Comparisons of the evolution of AMH values over time were restricted to the same patients, using paired tests (Wilcoxon).

A *p*-value of <0.05 was considered statistically significant. Tests were performed using the R® statistical software version 3.4.3. Figures were made using the ggplot2 package (R Core Team 2017, https://www.R-project.org/).

## Results

### Patient Characteristics

**Figure 1:**
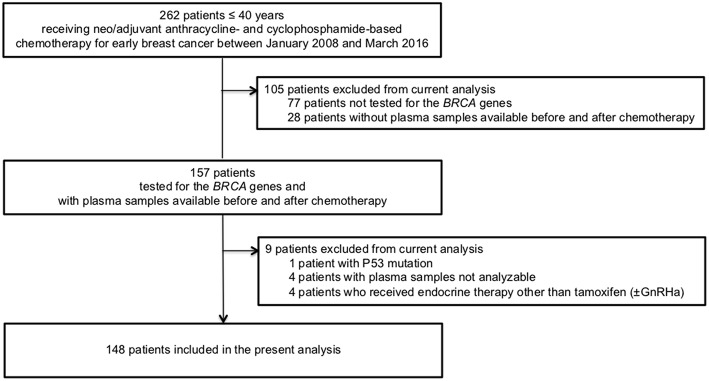
The flow diagram of participants. GnRHa, gonadotropin-releasing hormone agonists.

Between January 2008 and December 2016, out of 262 patients diagnosed at ≤ 40 years who underwent (neo)adjuvant chemotherapy for early breast cancer, 148 had plasma samples and *BRCA* mutational status available to be included in the present analysis (Figure 1).

Median age was 35 years (IQR 31.5–38; Table 1). A total of 127 (86%) patients received D in addition to FEC chemotherapy, 90 (61%) underwent endocrine therapy, and 35 (24%) had deleterious germline *BRCA* mutations.

**Table 1:** Baseline patients' and tumor characteristics.

**Patient characteristics**	**All patients (*n* = 148)**
Age at diagnosis, median [IQR]	35.5 [31.5–38]
AMH, median [IQR]	1.68 [1.00–3.30]
AMH, mean [SD]	2.55 [2.81]
Body mass index (kg/m^2^), median [IQR]	22.7 [21.0–26.2]
Smoker, n (%)	52 (35%)
**Genetic mutation**, ***n*** **(%)**	35 (24%)
*BRCA1*	22 (63%)
*BRCA2*	13 (37%)
**Tumor characteristics**, ***n*** **(%)**	
*Histological grade*	
Grade I	6 (4%)
Grade II	54 (36%)
Grade III	87 (59%)
Not available	1 (<1%)
*Hormone receptor positivity*	92 (62%)
Oestrogen receptor	90 (61%)
Progesteron receptor	65 (44%)
*HER2-positive*	27 (18%)
*Triple-negative*	52 (35%)
*Pathological nodal status positivity*	77 (52%)
*Tumor size (T)*	
T1	49 (33%)
T2	72 (49%)
T3	23 (15%)
T4	4 (3%)
**Surgical treatment**, ***n*** **(%)**	
Conservative	82 (55%)
Mastectomy	66 (45%)
**Adjuvant treatment**, ***n*** **(%)**	
Radiation therapy	144 (97%)
Endocrine therapy^a^	90 (61%)
Chemotherapy	148 (100%)
**Chemotherapy regimen**, ***n*** **(%)**	
3 FEC−3 D	127 (86%)
6 FEC	21 (14%)
**Endocrine therapy**, ***n*** **(%)**	
Tamoxifen	82 (91%)
Tamoxifen + GnRH agonists	8 (9%)
**Fertility history**, ***n*** **(%)**	
Pregnancy before treatment	129 (84%)
Childbirth before treatment	128 (84%)

a *2 patients with hormone receptor-positive tumors refused endocrine therapy*. *AMH, anti-mullerian hormone; IQR, interquartile range; SD, standard deviation; FEC, fluorouracil, epirubicin, cyclophosphamide; D, docetaxel; GnRH, gonadotropin-releasing hormone*.

### AMH Evolution Under Chemotherapy in the Whole Population

At baseline, median AMH level was 1.68 μg/L (IQR 1.00–3.30). Higher age was associated with significant lower AMH levels (*p* = 0.047; Figure S1).

One year after diagnosis (median 387 days, IQR 363–426), AMH levels drastically dropped to a median value of 0.06 μg/L (IQR 0–0.25; *p* < 0.0001). Three years after diagnosis (median 1,132 days, IQR 1,099–1,186), a slight but significant recovery of AMH value was observed with a median level of 0.17 μg/L (IQR 0.04–0.41; *p* < 0.0001; Figure S2).

### Impact of Taxanes

Baseline characteristics according to type of chemotherapy (FEC only vs. FEC-D) are reported in Table S1.

**Figure 2:**
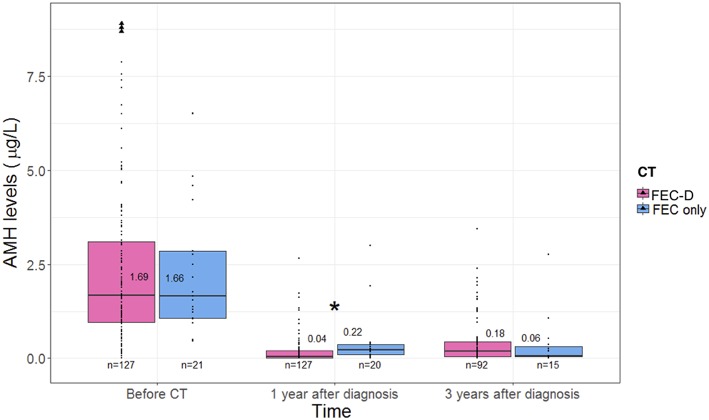
The evolution of anti-mullerian hormone levels according to type of chemotherapy. ^*^Statistical significant difference. AMH, anti-mullerian hormone; CT, chemotherapy; FEC, fluorouracil, epirubicin, cyclophosphamide; D, docetaxel.

At baseline, no difference in AMH levels was observed between patients treated with FEC only (1.66 μg/L, IQR 1.06–2.85) or FEC-D (1.69 μg/L, IQR 0.98–3.33; *p* = 0.83) (Figure 2).

One year after diagnosis, patients treated with FEC only had higher median AMH levels (0.22 μg/L, IQR 0.10–0.36) as compared to those who received FEC-D (0.04 μg/L, IQR 0.00–0.21; *p* = 0.0006), respectively.

Three years after diagnosis, no difference in median AMH levels was observed between patients treated with FEC only (0.06 μg/L, IQR 0.04–0.32) or FEC-D (0.18 μg/L, IQR 0.04–0.43; *p* = 0.47). Patients treated with FEC only had no recovery of AMH values between 1 and 3 years (0.22 vs. 0.06 μg/L, respectively, *p* = 0.81) while patients treated by FEC-D had a slight but significant recovery (0.04 vs. 0.18 μg/L, *p* < 0.0001).

### Impact of Endocrine Therapy

Baseline characteristics according to use of endocrine therapy (yes vs. no) are reported in Table S2. Among the 90 (61%) patients who received endocrine therapy, 82 (91%) underwent tamoxifen alone and 8 (9%) tamoxifen combined with GnRH agonists (Table 1). At the 3-year timepoint, all but three patients who started endocrine therapy were still under treatment.

**Figure 3:**
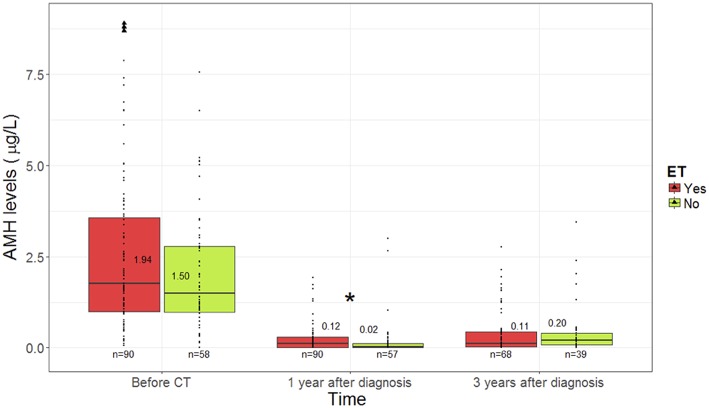
The evolution of anti-mullerian hormone levels according to use of endocrine therapy. ^*^Statistical significant difference. AMH, anti-mullerian hormone; ET, endocrine therapy.

At baseline, no difference in AMH values was observed between patients who received endocrine therapy (1.94 μg/L, IQR 1.01–3.76) and those who did not (1.50 μg/L, IQR 0.96–2.77; *p* = 0.17) (Figure 3).

One year after chemotherapy, patients under endocrine therapy had significantly higher AMH levels (0.12 μg/L, IQR 0.02–0.29) than those who did not receive this treatment (0.02 μg/L, IQR 0.00–0.12; *p* = 0.008).

No difference was observed 3 years after diagnosis, with comparable values of AMH for patients undergoing endocrine therapy (0.11 μg/L, IQR 0.03–0.43) or not (0.20 μg/L, 0.07–0.40; *p* = 0.22).

### Impact of Carrying a Deleterious Germline BRCA Mutation

Baseline characteristics according to *BRCA* mutational status (mutated vs. negative) are reported in Table S3. Among the 35 (24%) *BRCA*-mutated breast cancer patients, 22 and 13 harbored deleterious *BRCA1* or *BRCA2* mutations, respectively. Patients in the *BRCA*-mutated cohort were younger than those without mutation (*p* = 0.027).

**Figure 4:**
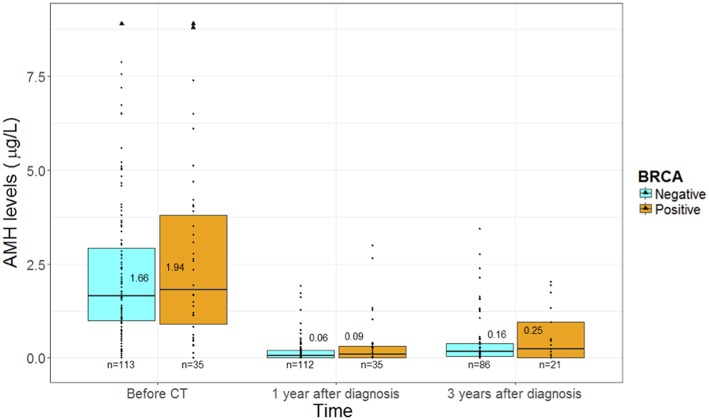
The evolution of anti-mullerian hormone levels according to *BRCA* mutational status. AMH, anti-mullerian hormone.

At baseline, no difference in AMH values was observed between *BRCA*-mutated (1.94 μg/L, IQR 0.98–3.96) and *BRCA*-negative (1.66 μg/L, IQR 1.00–3.02) patients (*p* = 0.53) (Figure 4).

One year after chemotherapy, AMH values drastically dropped in both groups to 0.09 μg/L (IQR 0.00–0.30) and 0.06 μg/L (IQR 0.00–0.21) in the *BRCA*-mutated and negative cohorts, respectively (*p* = 0.39).

Recovery at 3 years from diagnosis was observed in a similar extent in both groups with levels of 0.25 μg/L (IQR 0.02–0.95) and 0.16 μg/L (IQR 0.04–0.39) in the *BRCA*-mutated and negative cohorts, respectively (*p* = 0.43).

When the analyses were repeated to take into account the different age at diagnosis, similar results were shown with no difference in AMH levels between the *BRCA*-mutated and the *BRCA*-negative cohorts both at diagnosis and after treatment (Supplementary Data). Similarly, within the *BRCA*-mutated cohort, no impact of the type of *BRCA* mutation (*BRCA1* vs. *BRCA2*) was observed (Figure S3).

## Discussion

In this study, we assessed the ovarian reserve measured by AMH levels at baseline and up to 3 years after diagnosis in young early breast cancer patients treated with (neo)adjuvant anthracycline- and cyclophosphamide-based chemotherapy. While prior evidence exists on AMH levels following the use of endocrine therapy, this is the first study to our knowledge addressing the impact on patients' ovarian reserve of adding a taxane to anthracycline- and cyclophosphamide-based chemotherapy and the potential influence on treatment gonadotoxictiy of carrying a deleterious germline *BRCA* mutation. In early breast cancer patients receiving FEC chemotherapy, adding D appeared to negatively impact on their ovarian reserve in the short-term; no further detrimental effect was observed for endocrine therapy use and presence of a deleterious germline *BRCA* mutation.

Prior studies investigating treatment impact on patients' ovarian reserve showed that AMH decreases rapidly during the first cycles of chemotherapy reaching the lowest values at the end of systemic cytotoxic therapy (12–16). However, limited data on AMH values beyond 1 year following chemotherapy have been reported so far. Median AMH value at baseline in our study (1.68 μg/L) was in line with published data among non-cancer patients of similar age (median 2.3 μg/L) (17). With an estimated AMH value decline of 5.6%/year among non-cancer patients ≤ 40 years (17), the physiological decline in our population would have led to a median AMH value 3 years after diagnosis of 1.4 μg/L, which is far more elevated than the value observed (0.17 μg/L). Thus, our findings confirmed a deep and persistent impact of anthracycline- and cyclophosphamide-based chemotherapy on AMH levels up to 3 years after diagnosis. In addition, the specific features of our homogenous patient cohort provided a unique opportunity to investigate the influence on AMH levels of adding a taxane, using endocrine therapy as well as carrying a deleterious germline *BRCA* mutation.

A sequential treatment with anthracycline- and cyclophosphamide-based chemotherapy followed by a taxane is the current standard (neo)adjuvant chemotherapy regimen in young breast cancer patients (5). Despite being a widely used regimen since many years, the gonadotoxicity of such treatment remains controversial. While a prior meta-analysis showed no statistically significant increased risk of amenorrhea with the addition of a taxane (7), larger studies have recently shown a potential negative effect (18, 19). However, limited evidence exists on the actual and potential damage induced by taxanes on patients' ovarian reserve measured by AMH levels (20). Our findings, for the first time with a clear comparison between FEC only and FEC-D, provide some evidence on the potential increased gonadotoxic burden of administering a taxane following anthracycline- and cyclophosphamide-based chemotherapy. However, partial AMH recovery between 1 and 3 years after diagnosis was observed for FEC-D while a further decrease was shown for FEC only: hence, there was no difference between the two treatment options at 3 years. Notably, the dose of cyclophosphamide for patients treated with FEC only was approximately twice as compared to the one received by women who underwent FEC-D (6 vs. 3 cycles, respectively). As shown in animal models, taxanes appear to damage specifically the growing follicles with no apparent direct effect on primordial follicles (21). This specific gonadotoxic mechanism may explain our observation on the early (within 1 year) negative impact of taxanes on AMH levels. This is in contrast with cyclophosphamide that causes massive atresia of both primordial and growing follicles, with subsequent longer impact on women ovarian reserve reflected by poorer long-term AMH recovery (22).

All young women with hormone receptor-positive breast cancer are candidates to receive adjuvant endocrine therapy (5). For those who are previously exposed to chemotherapy, it is crucial to counsel them on the potential impact of this additional treatment on their ovarian reserve. Several studies have shown an increased risk of post-treatment amenorrhea when tamoxifen was administered after chemotherapy (7, 18, 19). On the contrary, the limited data on endocrine therapy gonadotoxicity measured by AMH levels did not show any difference between patients who received or not tamoxifen following chemotherapy (8, 13, 16, 23). Our findings confirm the lack of detrimental effect of endocrine therapy on patients' ovarian reserve. Interestingly, patients treated with tamoxifen had significantly higher AMH levels at 1 year as compared to those who received chemotherapy alone. Similarly, higher AMH values in patients treated with tamoxifen following chemotherapy has been also observed in two recent studies (8, 23), with one of them showing a faster AMH recovery between 3 and 6 months after the end of systemic cytotoxic therapy for women treated with endocrine therapy (23). Notably, when given concurrently with chemotherapy, preclinical studies have suggested a potential protective effect of tamoxifen against anticancer treatment gonadotoxicity, including of cyclophosphamide-based therapy (24). Taken together, although tamoxifen may cause perturbation in menstrual function after chemotherapy, the available evidence including our findings suggests the lack of detrimental effect on patients' ovarian reserve. Nevertheless, a proper oncofertility counseling in these women is particularly important and should take into account also the need to prolong such treatment up to 10 years after diagnosis (5), with their subsequent ovarian aging. Indeed, patients with hormone receptor-positive breast cancer have lower chances of post-treatment pregnancies as compared to women who are not candidates to endocrine therapy (8, 25). An international study is currently ongoing to investigate the safety of a temporary interruption of endocrine therapy after 18–30 months of treatment to allow a pregnancy (26).

More than 10% of breast cancer cases arising in young women are hereditary tumors related to the presence of a deleterious germline *BRCA* mutation (27, 28). Considering the high mutation rate in this patient population, current guidelines strongly support a genetic testing in all women diagnosed at a young age irrespectively of their family history (5). Preclinical and clinical evidence has suggested a potential negative impact of these mutations on female reproductive potential (29, 30). In breast cancer patients, three small studies investigated AMH levels in *BRCA*-mutated breast cancer patients but only at the time of diagnosis without data after chemotherapy (9, 10, 31). Therefore, a major unanswered concern in this setting is the potential increased gonadotoxicity risk of *BRCA*-mutated patients (29, 30). The only study that assessed chemotherapy-induced amenorrhea in this setting did not show any difference between *BRCA*-mutated and *BRCA*-negative patients (32). Our study is on the same line suggesting for the first time the lack of detrimental effect for carrying a deleterious germline *BRCA* mutation on chemotherapy-induced gonadal damage. Further studies in breast cancer patients with germline mutations in *BRCA* or other susceptibility genes are warranted to improve their oncofertility counseling in terms of both estimating the risk of gonadotoxicity with the proposed anticancer treatments as well as the efficacy and safety of fertility preservation procedures in this setting (10, 33).

Some limitations should be considered in the interpretation of our results. This analysis was conducted in a single center and included a relatively small cohort of patients who received FEC only chemotherapy or carried a deleterious germline *BRCA* mutation. A few post-chemotherapy plasma samples were missing and could not be assessed in the whole study cohort. Data on post-treatment pregnancies and menstrual function were not collected so that no correlation with AMH values could be performed. Nevertheless, despite these limitations, our study represents one of the largest AMH analyses and with the longest follow-up conducted so far among breast cancer patients; plasma samples were prospectively collected at predefined timepoints during treatment and oncologic follow-up up to 3 years after diagnosis in a homogenous cohort of women. In addition, our study provides answers on three relevant questions that are of crucial importance in everyday clinical practice to properly counsel these women on the gonadotoxicity of the proposed anticancer treatments. Future larger prospective studies are needed to address the clinical utility of this biomarker before its incorporation in routine clinical practice including its potential role as predictor of treatment-induced POI and infertility in young cancer patients.

## Conclusions

Our study confirmed the deep and prolonged adverse effect of chemotherapy on the ovarian reserve of young breast cancer patients with only partial recovery 3 years after treatment. We showed that adding D following anthracycline- and cyclophosphamide-based chemotherapy (FEC regimen) appeared to cause an early negative impact on their ovarian reserve. Endocrine therapy with tamoxifen and the presence of a deleterious germline *BRCA* mutation did not appear to further worsen chemotherapy gonadotoxicity. Altogether, although future larger collaborative efforts are needed to validate our results, we provide important data for improving the oncofertility counseling of young breast cancer patients.

## Data Availability

The datasets generated for this study are available on reasonable request to the corresponding author.

## Ethics Statement

The present study was approved by the local Institutional Review Board (registering order N°1807B). All patients signed a consent form allowing the conservation and study of their biological samples.

## Author Contributions

MLa, AP, and FC contributed to the conception and design of the study. CC and AP performed the analysis of all plasma samples. JL performed the statistical analysis. The results were interpreted by MLa, NO, JL, AP, and FC who also drafted the initial manuscript that was then revised critically for important intellectual content and approved by all the authors. All the authors contributed to collection and assembly of data.

### Conflict of Interest Statement

MLa served as a consultant for Teva and received honoraria from Theramex and Takeda outside the submitted work. FC received institutional research funds from Astra Zeneca and served as consultant for Astra Zeneca, Merck and BMS, outside of the submitted work. The remaining authors declare that the research was conducted in the absence of any commercial or financial relationships that could be construed as a potential conflict of interest.

## References

[1] Azim HA Partridge AH. Biology of breast cancer in young women. Breast Cancer Res. (2014) 16:427. 10.1186/s13058-014-0427-5 25436920 PMC4303229

[2] Partridge AH Hughes ME Warner ET Ottesen RA Wong Y-N Edge SB. Subtype-dependent relationship between young age at diagnosis and breast cancer survival. J Clin Oncol. (2016) 34:3308–14. 10.1200/JCO.2015.65.8013 27480155

[3] Lambertini M Goldrat O Clatot F Demeestere I Awada A. Controversies about fertility and pregnancy issues in young breast cancer patients: current state of the art. Curr Opin Oncol. (2017) 29:243–52. 10.1097/CCO.0000000000000380 28463857

[4] Peccatori FA Azim HA Jr. Orecchia R Hoekstra HJ Pavlidis N Kesic V Cancer, pregnancy and fertility: ESMO clinical practice guidelines for diagnosis, treatment and follow-up. Ann Oncol. (2013) 24 Suppl 6:vi160–70. 10.1093/annonc/mdt199 23813932

[5] Paluch-Shimon S Pagani O Partridge AH Abulkhair O Cardoso M-J Dent RA. ESO-ESMO 3rd international consensus guidelines for breast cancer in young women (BCY3). Breast. (2017) 35:203–17. 10.1016/j.breast.2017.07.017 28822332

[6] Oktay K Harvey BE Partridge AH Quinn GP Reinecke J Taylor HS. Fertility preservation in patients with cancer: ASCO clinical practice guideline update. J Clin Oncol. (2018) 36:1994–2001. 10.1200/JCO.2018.78.1914 29620997

[7] Zavos A Valachis A. Risk of chemotherapy-induced amenorrhea in patients with breast cancer: a systematic review and meta-analysis. Acta Oncol. (2016) 55:664–70. 10.3109/0284186X.2016.1155738 27105082

[8] Shandley LM Spencer JB Fothergill A Mertens AC Manatunga A Paplomata E. Impact of tamoxifen therapy on fertility in breast cancer survivors. Fertil Steril. (2017) 107:243–52.e5. 10.1016/j.fertnstert.2016.10.020 27887709 PMC5203952

[9] Titus S Li F Stobezki R Akula K Unsal E Jeong K. Impairment of BRCA1-related DNA double-strand break repair leads to ovarian aging in mice and humans. Sci Transl Med. (2013) 5:172ra21. 10.1126/scitranslmed.3004925 23408054 PMC5130338

[10] Lambertini M Goldrat O Ferreira AR Dechene J Azim HA Desir J. Reproductive potential and performance of fertility preservation strategies in BRCA-mutated breast cancer patients. Ann Oncol. (2018) 29:237–43. 10.1093/annonc/mdx639 29045555

[11] Partridge AH Ruddy KJ Gelber S Schapira L Abusief M Meyer M. Ovarian reserve in women who remain premenopausal after chemotherapy for early stage breast cancer. Fertil Steril. (2010) 94:638–44. 10.1016/j.fertnstert.2009.03.045 19409543

[12] Fréour T Barrière P Masson D. Anti-müllerian hormone levels and evolution in women of reproductive age with breast cancer treated with chemotherapy. Eur J Cancer. (2017) 74:1–8. 10.1016/j.ejca.2016.12.008 28135602

[13] Dezellus A Barriere P Campone M Lemanski C Vanlemmens L Mignot L. Prospective evaluation of serum anti-Müllerian hormone dynamics in 250 women of reproductive age treated with chemotherapy for breast cancer. Eur J Cancer. (2017) 79:72–80. 10.1016/j.ejca.2017.03.035 28463758

[14] Trapp E Steidl J Rack B Kupka MS Andergassen U Jückstock J. Anti-Müllerian hormone (AMH) levels in premenopausal breast cancer patients treated with taxane-based adjuvant chemotherapy–a translational research project of the SUCCESS A study. Breast. (2017) 35:130–5. 10.1016/j.breast.2017.07.007 28732324

[15] Perdrix A Saint-Ghislain M Degremont M David M Khaznadar Z Loeb A. Influence of adjuvant chemotherapy on anti-Müllerian hormone in women below 35 years treated for early breast cancer. Reprod Biomed Online. (2017) 35:468–74. 10.1016/j.rbmo.2017.06.005 28652099

[16] Anderson RA Mansi J Coleman RE Adamson DJA Leonard RCF. The utility of anti-Müllerian hormone in the diagnosis and prediction of loss of ovarian function following chemotherapy for early breast cancer. Eur J Cancer. (2017) 87:58–64. 10.1016/j.ejca.2017.10.001 29117576 PMC5733385

[17] Bentzen JG Forman JL Johannsen TH Pinborg A Larsen EC Andersen AN. Ovarian antral follicle subclasses and anti-mullerian hormone during normal reproductive aging. J Clin Endocrinol Metab. (2013) 98:1602–11. 10.1210/jc.2012-1829 23463653

[18] Silva C Caramelo O Almeida-Santos T Ribeiro Rama AC. Factors associated with ovarian function recovery after chemotherapy for breast cancer: a systematic review and meta-analysis. Hum Reprod. (2016) 31:2737–49. 10.1093/humrep/dew224 27664208

[19] Lambertini M Campbell C Bines J Korde LA Izquierdo M Fumagalli D. Adjuvant anti-HER2 therapy, treatment-related amenorrhea, and survival in premenopausal HER2-positive early breast cancer patients. J Natl Cancer Inst. (2019) 111:86–94. 10.1093/jnci/djy094 29878225 PMC6335113

[20] Anderson RA Themmen APN Al-Qahtani A Groome NP Cameron DA. The effects of chemotherapy and long-term gonadotrophin suppression on the ovarian reserve in premenopausal women with breast cancer. Hum Reprod. (2006) 21:2583–92. 10.1093/humrep/del201 16820385

[21] Lopes F Smith R Anderson RA Spears N. Docetaxel induces moderate ovarian toxicity in mice, primarily affecting granulosa cells of early growing follicles. Mol Hum Reprod. (2014) 20:948–59. 10.1093/molehr/gau057 25080441 PMC4172173

[22] Yuksel A Bildik G Senbabaoglu F Akin N Arvas M Unal F. The magnitude of gonadotoxicity of chemotherapy drugs on ovarian follicles and granulosa cells varies depending upon the category of the drugs and the type of granulosa cells. Hum Reprod. (2015) 30:2926–35. 10.1093/humrep/dev256 26466914

[23] Decanter C Cloquet M Dassonneville A D'Orazio E Mailliez A Pigny P. Different patterns of ovarian recovery after cancer treatment suggest various individual ovarian susceptibilities to chemotherapy. Reprod Biomed Online. (2018) 36:711–8. 10.1016/j.rbmo.2018.02.004 29523398

[24] Piasecka-Srader J Blanco FF Delman DH Dixon DA Geiser JL Ciereszko RE. Tamoxifen prevents apoptosis and follicle loss from cyclophosphamide in cultured rat ovaries. Biol Reprod. (2015) 92:132. 10.1095/biolreprod.114.126136 25833159 PMC4645984

[25] Lambertini M Moore HCF Leonard RCF Loibl S Munster P Bruzzone M. Gonadotropin-releasing hormone agonists during chemotherapy for preservation of ovarian function and fertility in premenopausal patients with early breast cancer: a systematic review and meta-analysis of individual patient-level data. J Clin Oncol. (2018) 36:1981–90. 10.1200/JCO.2018.78.0858 29718793 PMC6804855

[26] Pagani O Ruggeri M Manunta S Saunders C Peccatori F Cardoso F. Pregnancy after breast cancer: are young patients willing to participate in clinical studies? Breast. (2015) 24:201–7. 10.1016/j.breast.2015.01.005 25662412

[27] Rosenberg SM Ruddy KJ Tamimi RM Gelber S Schapira L Come S. BRCA1 and BRCA2 mutation testing in young women with breast cancer. JAMA Oncol. (2016) 2:730–6. 10.1001/jamaoncol.2015.5941 26867710 PMC5002892

[28] Copson ER Maishman TC Tapper WJ Cutress RI Greville-Heygate S Altman DG. Germline BRCA mutation and outcome in young-onset breast cancer (POSH): a prospective cohort study. Lancet Oncol. (2018) 19:169–80. 10.1016/S1470-2045(17)30891-4 29337092 PMC5805863

[29] Lambertini M Goldrat O Toss A Azim HA Peccatori FA Ignatiadis M. Fertility and pregnancy issues in BRCA-mutated breast cancer patients. Cancer Treat Rev. (2017) 59:61–70. 10.1016/j.ctrv.2017.07.001 28750297

[30] Peccatori FA Mangili G Bergamini A Filippi F Martinelli F Ferrari F. Fertility preservation in women harboring deleterious BRCA mutations: ready for prime time? Hum Reprod. (2018) 33:181–7. 10.1093/humrep/dex356 29207007

[31] Gunnala V Fields J Irani M D'Angelo D Xu K Schattman G. BRCA carriers have similar reproductive potential at baseline to noncarriers: comparisons in cancer and cancer-free cohorts undergoing fertility preservation. Fertil Steril. (2019) 111:363–71. 10.1016/j.fertnstert.2018.10.014 30527950

[32] Valentini A Finch A Lubinski J Byrski T Ghadirian P Kim-Sing C Chemotherapy-induced amenorrhea in patients with breast cancer with a BRCA1 or BRCA2 mutation. J Clin Oncol. (2013) 31:3914–9. 10.1200/JCO.2012.47.7893 23980083 PMC3805929

[33] Turan V Bedoschi G Emirdar V Moy F Oktay K. Ovarian stimulation in patients with cancer: impact of letrozole and BRCA mutations on fertility preservation cycle outcomes. Reprod Sci. (2018) 25:26–32. 10.1177/1933719117728800 28874104

