# Supplementary material for: Impact of Taxanes, Endocrine Therapy, and Deleterious Germline BRCA Mutations on Anti-müllerian Hormone Levels in Early Breast Cancer Patients Treated With Anthracycline- and Cyclophosphamide-Based Chemotherapy
Source: Front Oncol. 2019 Jul 12;9:575. doi: 10.3389/fonc.2019.00575 (PMC6640206; doi:10.3389/fonc.2019.00575)
Supplement: Supplementary file 1 [file Table_1.DOCX]

**Additional file 1: Table S1.** Baseline patients’ and tumor characteristics according to type of chemotherapy.

| **Patient characteristics** | **FEC only**  **(n=21)** | **FEC-D**  **(n=127)** | **p** |
| --- | --- | --- | --- |
| Age at diagnosis, median [IQR]  AMH, median [IQR]  AMH, mean [SD]  Body mass index (kg/m²), median [range]  Smoker, n (%)  Genetic mutation, n (%)  *BRCA1*  *BRCA2*  Tumor characteristics, n (%)  *Histological grade*  Grade I  Grade II  Grade III  Not available  *Hormone receptor positivity*  Oestrogen receptor  Progesteron receptor  *HER2-positive*  *Triple-negative*  *Pathological nodal status positivity*  *Tumor size (T)*  T1  T2  T3  T4  Surgical treatment, n (%)  Conservative  Mastectomy  Adjuvant treatment, n (%)  Radiation therapy  Endocrine Therapyᵃ  Chemotherapy  Chemotherapy regimen, n (%)  3 FEC - 3 D  6 FEC  Endocrine therapy, n (%)  Tamoxifen  Tamoxifen + GnRH agonists  Fertility history  Pregnancy before treatment  Child birth before treatment | 35 [32-38]  1.66 [1.06-2.85]  2.39 [1.88]  22.0 [20.7-25.9]  10 (48%)  4 (19%)  1 (25%)  3 (75%)  1 (5%)  7 (33%)  13 (62%)  0  17 (81%)  16 (76%)  12 (57%)  1 (5%)  3 (14%)  2 (9%)  8 (38%)  13 (62%)  0  0  10 (48%)  11 (52%)  20 (95%)  16 (76%)  21 (100%)  0 (0%)  21 (100%)  11 (69%)  5 (31%)  19 (91%)  19 (91%) | 36 [31-38]  1.69 [0.98-3.33]  2.57 [2.94]  23.0 [21.1-26.4]  42 (33%)  31 (24%)  21 (68%)  10 (32%)  5 (4%)  47 (37%)  74 (58%)  1 (<1%)  75 (59%)  74 (58%)  53 (42%)  26 (20%)  49 (39%)  75 (35%)  41 (32%)  59 (46%)  23 (18%)  4 (3%)  72 (57%)  55 (43%)  124 (98%)  74 (82%)  127 (100%)  127 (100%)  0 (0%)  71 (96%)  3 (4%)  105 (83%)  104 (82%) | 0.72  0.83  0.42  0.3  0.78  0.92  0.09  0.19  0.28  0.13  0.05  <0.0001  0.14  0.44  0.46  0.19  0.005    0.39  0.22  0.47 |

ᵃ 1 patient receiving FEC-only regimen and 1 patient receiving 3FEC-3D regimen with hormone receptor-positive tumors refused endocrine therapy

Abbreviations: AMH, anti-mullerian hormone; FEC, fluorouracil, epirubicin, cyclophosphamide; D, docetaxel; IQR, interquartile range; SD, standard deviation; GnRH, gonadotropin-releasing hormone.

**Additional file 2: Table S2.** Baseline patients’ and tumor characteristics according to use of endocrine therapy.

| **Patient characteristics** | **Yes ET**  **(n=90)** | **No ET**  **(n=58)** | **p** |
| --- | --- | --- | --- |
| Age at diagnosis, median [IQR]  AMH, median [IQR]  AMH, mean [SD]  Body mass index (kg/m²), median [IQR]  Smoker, n (%)  Genetic mutation, n (%)  *BRCA1*  *BRCA2*  Tumor characteristics, n (%)  *Histological grade*  Grade I  Grade II  Grade III  Not available  *Hormone receptor positivity*  Oestrogen receptor  Progesteron receptor  *HER2-positive*  *Triple-negative*  *Pathological nodal status positivity*  *Tumor size (T)*  T1  T2  T3  T4  Surgical treatment, n (%)  Conservative  Mastectomy  Adjuvant treatment, n (%)  Radiation therapy  Endocrine Therapyᵃ  Chemotherapy  Chemotherapy regimen, n (%)  3 FEC - 3 D  6 FEC  Endocrine therapy, n (%)  Tamoxifen  Tamoxifen + GnRH agonists  Fertility history, n (%)  Pregnancy before treatment  Child birth before treatment | 36 [32-38]  1.94 [1.01-3.76]  2.88 [3.30]  22.4 [20.9-26.3]  30 (33%)  15 (17%)  4 (27%)  11 (63%)  6 (4%)  44 (49%)  40 (44%)  0  90 (100%)  88 (98%)  63 (70%)  23 (26%)  0 (0%)  50 (56%)  29 (32%)  46 (51%)  15 (17%)  0  40 (45%)  50 (55%)  88 (98%)  90 (100%)  90 (100%)  74 (82%)  16 (18%)  82(91%)  8(9%)  75 (83%)  75 (83%) | 35 [31-38]  1.50 [0.96-2.77]  2.03 [1.68]  23.0 [21.1-26.7]  22 (38%)  20 (35%)  18 (90%)  2 (10%)  0 (0%)  10 (18%)  47 (81%)  1 (<1%)  2 (3.5%)  2 (3.5%)  2 (3.5%)  4 (7%)  52 (90%)  27 (47%)  20 (35%)  26 (45%)  8 (14%)  4 (7%)  42 (72%)  16 (28%)  56 (97%)  0 (0%)  58 (100%)  52 (90%)  4 (10%)  0  0  49 (85%)  48 (83%) | 0.32  0.17  0.59  0.69  0.02  <0.0001  <0.0001  <0.0001  0.004  NA  0.37  0.08  0.001  0.64  0.19  NA  0.44  0.61 |

ᵃ 2 patients with hormone receptor-positive tumors refused endocrine therapy

Abbreviations: AMH, anti-mullerian hormone; ET, endocrine therapy; IQR, interquartile range; SD, standard deviation; FEC, fluorouracil, epirubicin, cyclophosphamide; D, docetaxel; GnRH, gonadotropin-releasing hormone.

**Additional file 3: Table S3.** Baseline patients’ and tumor characteristics according to *BRCA* mutational status.

| **Patient characteristics** | ***BRCA*-mutated (n=35)** | ***BRCA-*negative (n=113)** | **p** |
| --- | --- | --- | --- |
| Age at diagnosis, median [range]  AMH, median [IQR]  AMH, mean [SD]  Body mass index (kg/m²), median [range]  Smoker, n (%)  Genetic mutation, n (%)  *BRCA1*  *BRCA2*  Tumor characteristics, n (%)  *Histological grade*  Grade I  Grade II  Grade III  Not available  *Hormone receptor positivity*  Oestrogen receptor  Progesteron receptor  *HER2-positive*  *Triple-negative*  *Pathological nodal status positivity*  *Tumor size (T)*  *T1*  *T2*  *T3*  *T4*  Surgical treatment, n (%)  Conservative  Mastectomy  Adjuvant treatment, n (%)  Radiation therapy  Endocrine Therapyᵃ  Chemotherapy  Chemotherapy regimen, n (%)  3 FEC - 3 D  6 FEC  Endocrine therapy, n (%)  Tamoxifen  Tamoxifen + GnRH agonists  Fertility history, n (%)  Pregnancy before treatment  Child birth before treatment | 34 [31-36]  1.94 [0.98-3.96]  2.82 [2.98]  22.0 [20.3-26.3]  13 (37%)  35 (100%)  22 (63%)  13 (37%)  0  6 (17%)  29 (83%)  0  15 (43%)  15 (43%)  6 (17%)  1 (3%)  19 (54%)  17 (49%)  14 (40%)  16 (46%)  4 (11%)  1 (3%)  16 (46%)  19 (54%)  33 (94%)  15 (43%)  35 (100%)  31 (89%)  4 (11%)  14 (93%)  1 (7%)  29 (83%)  29 (83%) | 36 [33-39]  1.66 [1.00-3.02]  2.46 [2.76]  23.1[20.7-26.5]  39 (34%)  0  0  0  6(5%)  48 (43%)  58 (51%)  1 (<1%)  77 (68%)  75 (67%)  59 (52%)  26 (23%)  33 (29%)  60 (53%)  35 (31%)  56 (50%)  19 (16%)  3 (3%)  66 (58%)  47 (42%)  111 (98%)  75 (67%)  113 (100%)  96 (85%)  17 (15%)  68 (92%)  7 (8%)  95 (84%)  94 (83%) | 0.03  0.53  0.48  0.93  0.004  0.01  0.02  0.0005  0.005  0.01  0.78  1.24  0.19  0.24  0.002  0.78  0.19  0.36  0.52 |

ᵃ 2 *BRCA*-negative patients with hormone receptor-positive tumors refused endocrine therapy

Abbreviations: AMH, anti-mullerian hormone; IQR, interquartile range; SD, standard deviation; FEC, fluorouracil, epirubicin, cyclophosphamide; D, docetaxel; GnRH, gonadotropin-releasing hormone.

**Additional file 4: Figure S1.** Anti-mullerian hormone (AMH) levels by age at the time of breast cancer diagnosis in the whole study cohort.


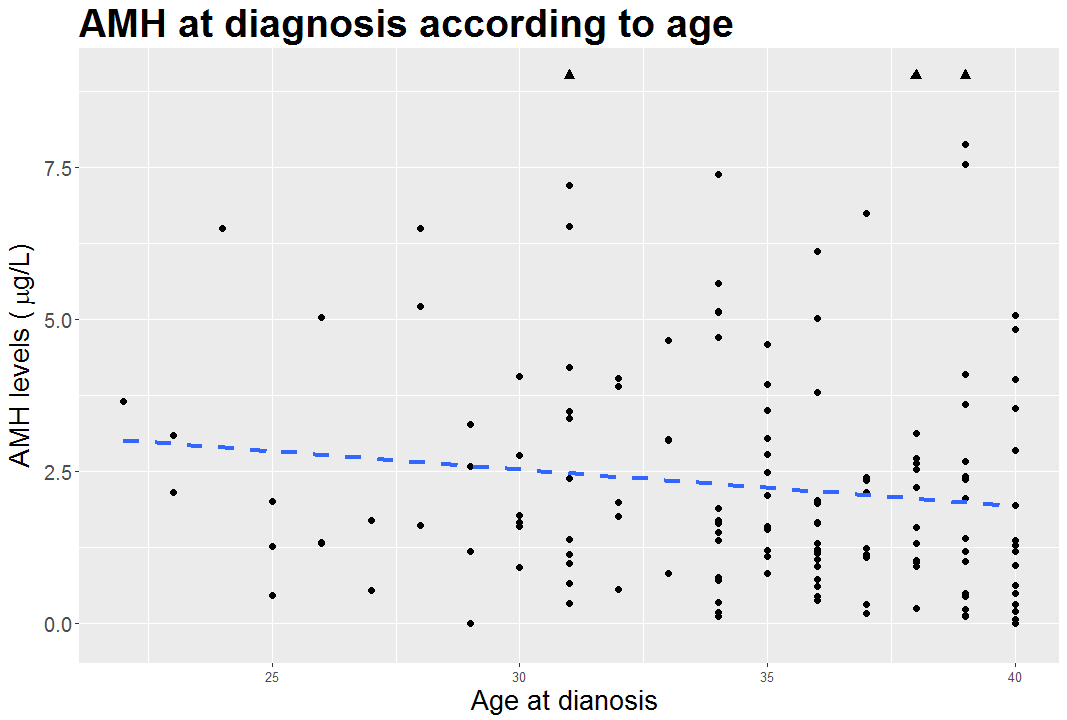


**Additional file 5: Figure S2.** The evolution of anti-mullerian hormone (AMH) levels in the whole study cohort.

**
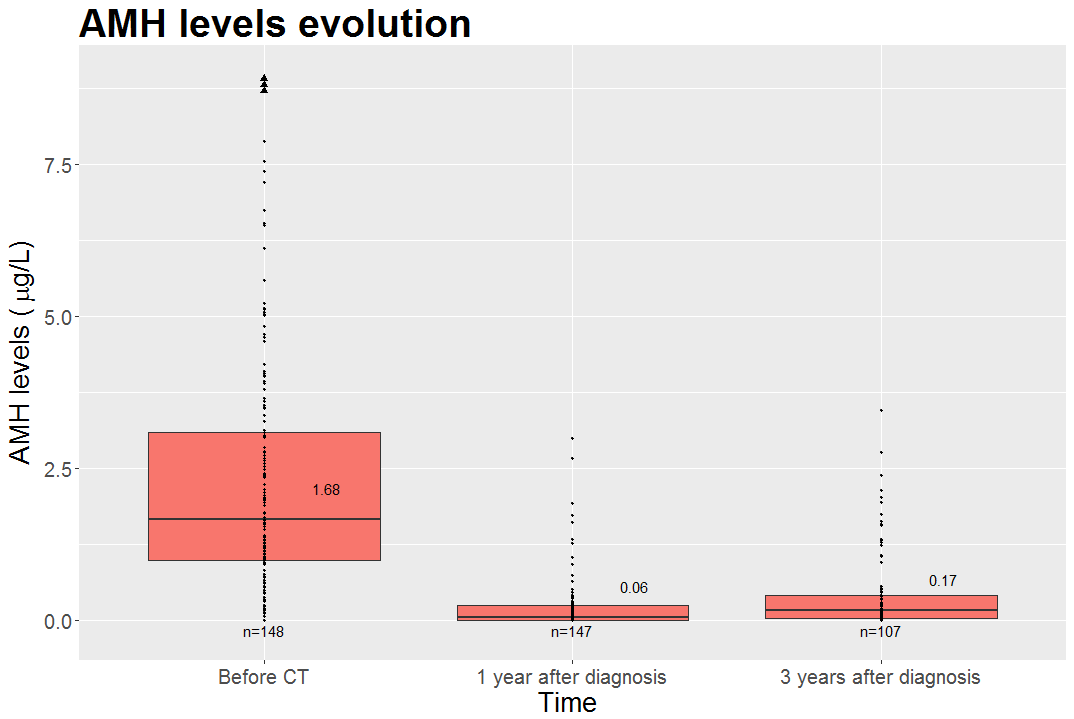
**

**Additional file 6: Figure S3.** The evolution of anti-mullerian hormone (AMH) levels in the whole study cohort according to presence/absence of *BRCA* mutation and the type of mutation (*BRCA1* vs. *BRCA2*).

**
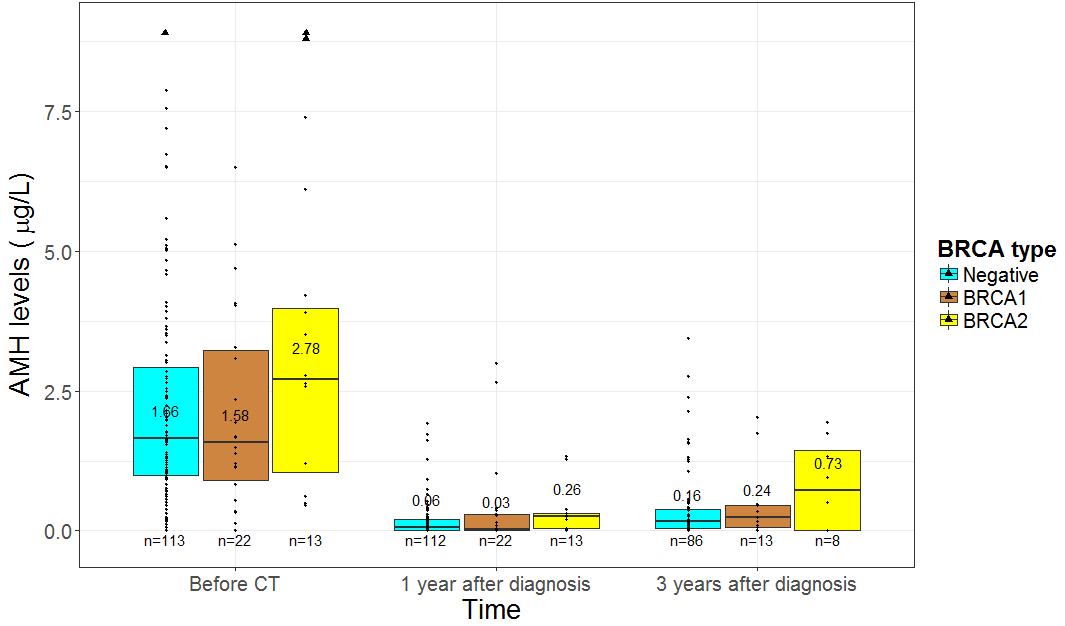
**

Since age was different in the *BRCA*-mutated and *BRCA*-negative cohorts, we investigated the presence of a potential interaction between age and *BRCA* mutational status regarding baseline anti-mullerian hormone (AMH) levels. Using a linear regression method, no interaction between these variables was observed.

In addition, we randomly constructed a paired-analysis based on age between *BRCA*-mutated and *BRCA*-negative patients to then compare baseline and post-treatment AMH values. Three different paired-analyses were made, and none reported a difference on AMH values at any time between the *BRCA*-mutated and *BRCA*-negative cohorts.
